# “Homeopathy is not placebo effect”: proof of the scientific evidence for homeopathy

**DOI:** 10.1590/1806-9282.20231438

**Published:** 2024-05-03

**Authors:** Marcus Zulian Teixeira

**Affiliations:** 1 Universidade de São Paulo, Hospital das Clínicas, Faculty of Medicine, Department of Psychiatry – São Paulo (SP), Brazil.

Homeopathy has been a medical practice recognized worldwide for more than two centuries, providing care, teaching, and research activities in several health institutions and medical schools. It employs a clinical approach based on non-conventional and complementary scientific principles (principle of therapeutic similitude, homeopathic pathogenetic experimentation, and use of dynamized doses and individualized medicines), with the aim of awakening a curative response from the body against its own disorders or diseases^1^.

Homeopathy proposes to understand and treat the sick-disease binomial according to a vitalist, globalizing, and humanist anthropological approach, valuing the different aspects of the sick individuality (mental, general, and physical) and contributes to maintain health and organic homeostasis, acting as a therapeutic alternative for various health disorders^2,3^.

However, to achieve this objective, homeopathic therapy must be well conducted and follow the epistemological premises of the homeopathic model^1^, among which include applying therapeutic similitude/similarity between the set of signs and symptoms of the sick individual (characteristic symptomatic totality of the sick-disease binomial) and the set of pathogenetic signs and symptoms caused by the medicine in the healthy individual (homeopathic pathogenetic experimentation), meaning individualized homeopathic treatment.

Several double-blind and placebo-controlled randomized clinical trials (RCTs) and their systematic reviews with meta-analyses which disrespected this therapeutic individualization by administering the same medication to different individuals with the same disease did not show significant results compared with placebo, as they violated scientific rationality of the homeopathic model^1,4,5^.

On the contrary, as homeopathy is based on premises different from those used by conventional medical practice, it is often the target of criticism and attacks by individuals who systematically disregard homeopathic assumptions and any scientific evidence that proves them, as they have a denialist and biased stance which prevents a correct and prejudice-free analysis. In reality, they are pseudoskeptics masquerading as pseudoscientists^6,7^.

To enlighten doctors, researchers, health professionals, and the general public, demystifying culturally ingrained dogmatic positions and the pseudoskeptical fallacies that “there is no scientific evidence for homeopathy” and that “homeopathy is placebo effect,” the Technical Chamber for Homeopathy (TC-Homeopathy) of the Regional Medical Council of the State of São Paulo (Cremesp), in 2017, developed the *Special Dossier: “Scientific Evidence for Homeopathy,”* available in three independent editions (online in Portuguese and English; printed in Portuguese) in the scientific journal *Revista de Homeopatia* (*São Paulo*). In 2023, the dossier was published in Spanish in the *La Homeopatía de México* journal in an edition commemorating its 90th anniversary^8–10^.

The respective dossier was composed of nine narrative reviews of research on several fields of medical science (historical, social, medical education, pharmacological, basic, clinical, patient safety, and pathogenetic experimentation) and two randomized clinical trials developed by TC-Homeopathy members, encompassing hundreds of scientific articles describing experimental and clinical studies, and seeks to highlight the state of the art of homeopathic research^8–10^.

To prove and expand this scientific evidence for homeopathy, on September 25, 2023^11^, we published an electronic book (e-book) in Portuguese “*Homeopatia não é efeito placebo*”: *comprovação das evidências científicas da homeopatia* (“*Homeopathy is not placebo effect*”: *proof of the scientific evidence for homeopathy*), indexed and made available in the Virtual Health Library (VHL-LILACS-BIREME)^12,13^, updating knowledge in the area in 13 interactive chapters. In addition to elucidating the epistemological premises of the homeopathic model in detail, the study describes data and bibliographic references in the information continuum, as well as the different areas of basic and clinical research in homeopathy which endorse homeopathic practice and treatment.

The study discusses various topics related to research in homeopathy, covering everything from homeopathic clinical epidemiology to the pseudoskeptical and pseudoscientific strategies used in attacks on homeopathy, including the panorama of research in homeopathy (databases), the pharmacological basis of the principle of similitude, experimental studies in biological models, randomized controlled clinical trials, systematic reviews and meta-analyses, and observational studies, among others^12,13^.

In the chapter “Homeopatia” (“Homeopathy”), the scientific evidence of homeopathic assumptions is described in general databases, discussing the epistemological premises of the homeopathic model in detail (principle of therapeutic similitude, homeopathic pathogenetic experimentation, and use of dynamized doses and individualized medicines)^1^ and providing the reader with an overview of treatment and clinical practice in homeopathy.

In “Epidemiologia clínica em homeopatia” (“Clinical epidemiology in homeopathy”), after a general review of the principles of clinical epidemiology and the types of epidemiological studies used to evaluate the efficacy and clinical effectiveness of conventional treatments, the premises and principles of homeopathic clinical epidemiology are described, as well as the types of epidemiological studies in homeopathy^14^. As we initially emphasized, the epistemological premise of individualized homeopathic treatment in the face of the characteristic symptomatic totality of the patient–disease binomial is a *sine qua non* condition for the ultra-diluted homeopathic medicine to be able to stimulate a significant curative response against its own disorders^1,4,5^. Failure to do so is a serious flaw in the design of homeopathic clinical trials of high methodological quality^14^.

In addition to the general databases, the various databases that group homeopathic experimental studies into biological and physicochemical models are described (“Homeopathy Basic Research Experiments database,” “HomVetCR database,” and “PROVINGS.INFO database”) in the chapter “Panorama da pesquisa em homeopatia—Bancos de dados” (“Overview of homeopathy research—Databases”), as well as homeopathic clinical epidemiological studies of all types (“Clinical Outcome Research in Homeopathy,” “Homeopathic Intervention Studies,” and “CAM-QUEST databases”). In these databases, readers will be able to see the wide range of studies indexed in the areas of basic and clinical research in homeopathy, with proposals for bibliographic surveys exemplified in each chapter of the study.

Next, the principle of therapeutic similitude is approached according to the homeopathic model and modern pharmacology in the chapter “Fundamentação farmacológica do princípio da similitude” (“Pharmacological basis of the principle of similitude”), describing hundreds of experimental and clinical studies that substantiate the curative response (vital reaction) of homeopathic treatment in accordance with the rebound effect of modern drugs (paradoxical reaction of the organism). Furthermore, it describes the proposal to use modern drugs according to the therapeutic similarity, using the rebound effect of drugs in a therapeutic way^15–17^.

In the field of basic research in homeopathy, the chapter “Estudos experimentais em modelos biológicos (in vitro, em vegetais e em animais)” [“Experimental studies in biological models (in vitro, in plants and animals)”] describes hundreds of controlled experimental studies in cells, plants, and animals, demonstrating the superiority of the effect of homeopathic medicine compared with control groups and showing through systematic reviews and meta-analyses that “homeopathy is not placebo effect”^18–20^.

In the field of clinical research in homeopathy, the chapter “Ensaios clínicos controlados randomizados (RCTs)” [“Randomized controlled clinical trials (RCTs)”] describes dozens of randomized, double-blind, placebo-controlled clinical trials (level of evidence 1B) with good methodological quality, which demonstrate the effectiveness of homeopathic treatment compared with placebo. Increasing the level of evidence of the clinical effectiveness of homeopathy (1A), four chapters address systematic reviews of RCTs, global (any clinical indication) and specific (specific clinical indication), with and without meta-analyses.

Then in the chapter “Revisões sistemáticas e relatórios globais com resultados positivos da homeopatia perante placebo” (“Systematic reviews and global reports with positive results of homeopathy compared to placebo”), five global systematic reviews with meta-analyses (and one global report) that demonstrated the superiority of homeopathic treatment over placebo are described. On the contrary, the studies that brought negative results of homeopathy compared with placebo are presented in the chapter “Revisões sistemáticas e relatórios globais com resultados negativos da homeopatia perante placebo (Falhas metodológicas)” [“Systematic reviews and global reports with negative results of homeopathy compared to placebo (Methodological flaws)”], including two global systematic reviews, one with a meta-analysis and the other without, and a global report, highlighting their numerous biases and methodological flaws, presented in several reanalyses published later (post hoc analyses).

Confirming these post hoc analyses, on October 7, 2023, a systematic review of global meta-analyses of RCTs was published demonstrating that “the quality of evidence for positive effects of homeopathy beyond placebo was high for individualised homeopathy and moderate for non individualised homeopathy” and that “there was no support for the alternative hypothesis of no outcome difference between homeopathy and placebo”^21^.

The chapter “Revisões sistemáticas para condições clínicas específicas” (“Systematic reviews for specific clinical conditions”) presents a description of specific systematic reviews that demonstrated the superiority of homeopathy over placebo, in various clinical conditions, with meta-analyses (allergic rhinitis, acute childhood diarrhea, postoperative ileus, and attention deficit hyperactivity disorder) and without meta-analyses (otitis acute media, postoperative inflammation, psychiatric disorders, and rheumatic diseases).

Then, in the chapter “Estudos observacionais” (“Observational studies”), we mainly discuss analytical observational studies (level of evidence 2B), describing robust cohort studies that present important information about the effectiveness and cost-effectiveness of homeopathic treatment in thousands of patients, both in the long term and in different clinical conditions^22–25^.

The final chapter “Estratégias pseudocéticas e pseudocientíficas usadas em ataques à homeopatia” (“Pseudoskeptical and pseudoscientific strategies used in attacks on homeopathy”) discusses pseudoskepticism and pseudoscience, describing the indicative signs of pseudoskepticism (false skepticism or pathological skepticism) in detail, which are topics of fundamental importance to unmask individuals who systematically maintain a denialist and dogmatic stance against homeopathy (pseudoskeptics and pseudoscientists), disregarding the countless existing scientific evidence which was presented in detail in the various chapters of this e-book^6,7^.

As we reiterate throughout the study, despite the difficulties and limitations that exist in developing research in homeopathy, both due to methodological aspects and the lack of institutional and financial support, the set of experimental and clinical studies described is indisputable proof that “there is scientific evidence for homeopathy” and that “homeopathy is not placebo effect,” contrary to falsely disseminated prejudice^6,7^. However, new studies must continue to be developed to improve clinical practice and elucidate characteristic aspects of the homeopathic paradigm.

Acting as an integrative and complementary therapy to other specialties, homeopathy can add efficacy, effectiveness, efficiency, and safety to medical practice, acting in a curative and preventive manner, reducing symptomatic manifestations and the predisposition to falling ill, with low cost and minimal adverse events, and helping physicians to fulfill their “high and *only* mission, which is to restore the sick to healthy, to cure, as it is termed” (Samuel Hahnemann, *Organon of medicine*, § 1).
